# Supplementary material for: Synthesis and Characterization of Coordination Compound [Eu(µ2-OC2H5)(btfa)(NO3)(phen)]2phen with High Luminescence Efficiency
Source: Nanomaterials (Basel). 2022 Aug 14;12(16):2788. doi: 10.3390/nano12162788 (PMC9415948; doi:10.3390/nano12162788)
Supplement: Supplementary file 1 [file nanomaterials-12-02788-s001.zip › nanomaterials-1787435-supplementary.pdf]

Supplementary Materials

Synthesis and Characterization of Coordination Compound [Eu(μ2-OC2H5)(btfa)(NO3)(phen)]2phen with High Luminescence Efficiency

Ion P. Culeac, Victor I. Verlan, Olga T. Bordian, Vera E. Zubareva, Mihail S. Iovu, Ion I. Bulhac, Nichita A. Siminel, Anatolii V. Siminel, Geanina Mihai and Marius Enachescu

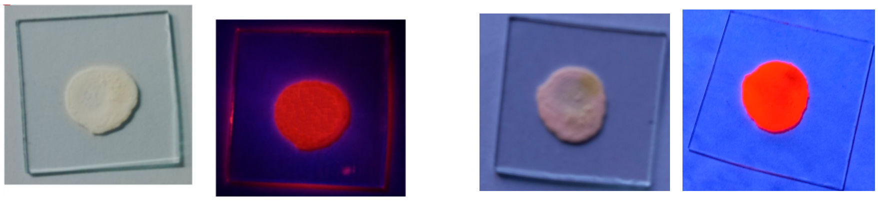

a *b*

Figure S1. Photographic images, representing the powder sample **kept in the** air over a three year period: (a) the images taken in 2018; and (b) the images taken in 2022. Each left image in the pair represents the sample under day-light illumination; the right image - the sample under blue-light irradiation. Apparently, there is no evident difference in the brightness of the luminescence.

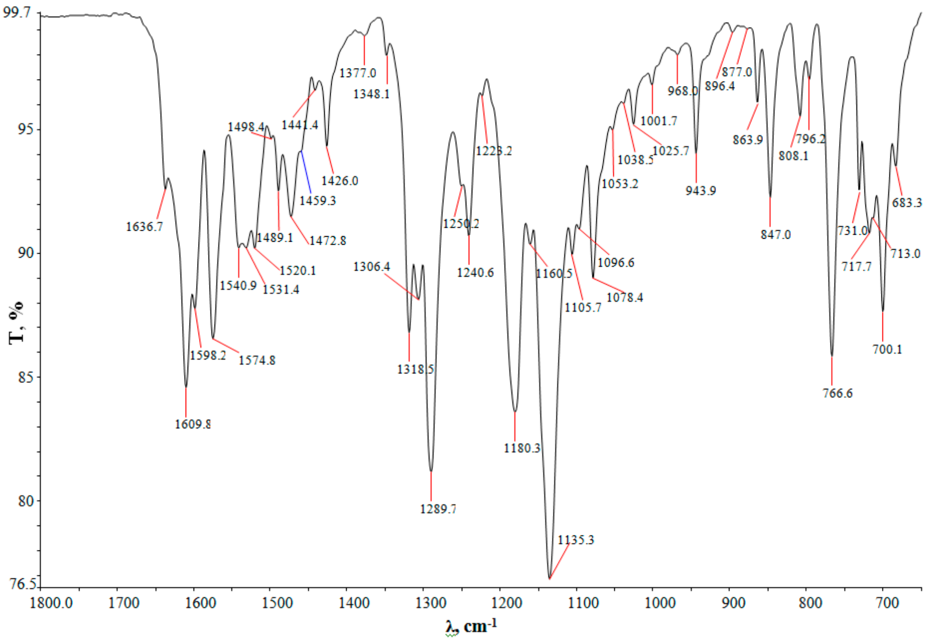

Figure S2. The IR spectrum of the complex [Eu(μ_2-OC_2H_5)(btfa)(NO_3)(phen)]$_2$phen.

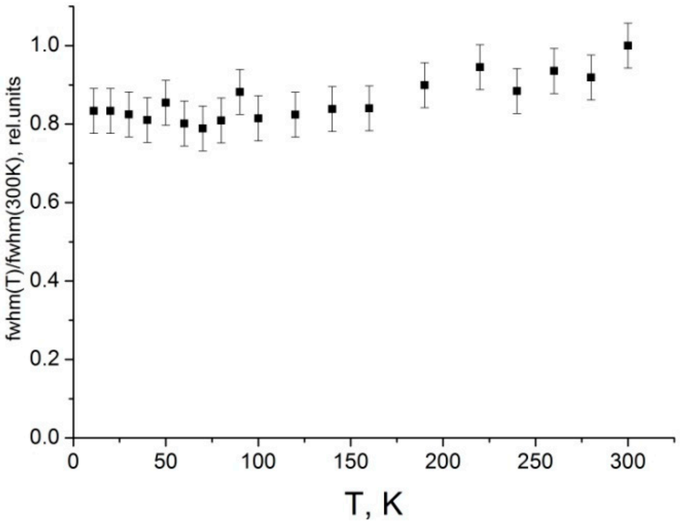

Figure S3. Illustration of narrowing trend of the $^5D_0$-$^7F_0$ bands with cooling down the sample.
